# Development and Verification of a Suspension-Based TXRF Method for Chromium Determination in Feed and Fecal Samples Containing Chromic Oxide as an External Digestibility Marker

**DOI:** 10.3390/molecules31091522

**Published:** 2026-05-03

**Authors:** Christina Tzvetkova, Lidia Ivanova, Albena Detcheva, Antonina Kovacheva, Miroslav Simeonov, Irina Karadjova

**Affiliations:** 1 Institute of General and Inorganic Chemistry, Bulgarian Academy of Sciences, Acad. G. Bontchev Str. Bl. 11, 1113 Sofia, Bulgaria; hrisi@svr.igic.bas.bg (C.T.); lidia@svr.igic.bas.bg (L.I.); albena@svr.igic.bas.bg (A.D.); akovacheva@svr.igic.bas.bg (A.K.); 2 National Centre of Excellence Mechatronics and Clean Technologies, 8 bul. Kliment Ohridski, 1756 Sofia, Bulgaria; 3 Agricultural Institute, Agricultural Academy, 6000 Stara Zagora, Bulgaria; msimeonov78@abv.bg

**Keywords:** chromium, chromic oxide, Cr_2_O_3_, external marker, feed and fecal samples, TXRF analysis, ICP-OES analysis

## Abstract

In the present study, a rapid method based on total reflection X-ray fluorescence (TXRF) was developed to determine chromium in feed and fecal samples, containing chromic oxide. The method uses suspension sample preparation with gallium as an internal standard and does not require chemical reagents or complete sample digestion. Key parameters affecting performance, such as particle size, sample concentration, and acquisition time, were optimized to ensure stable signals and reliable quantification. The method showed a limit of quantification of 24 µg g^−1^ for Cr_2_O_3_ and good precision (relative standard deviations of 3–7% for both feed and fecal samples), at Cr_2_O_3_ concentrations in the range of 20–50 mg kg^−1^. These performance characteristics meets the requirements for digestibility studies. It requires only small sample quantities with minimal preparation. The developed method is suitable for routine analysis, particularly in studies generating large numbers of samples. The accuracy of the method was confirmed through agreement with results obtained using an independent method based on inductively coupled plasma optical emission spectrometry (ICP-OES) after acid digestion. The scatter plot analysis of the results obtained by both methods showed a linear regression line with a slope of 0.978 and a correlation coefficient (R^2^) of 0.9559, indicating good agreement. The *p*-value from the paired *t*-test performed was greater than 0.05, suggesting that the observed differences between paired measurements are not statistically significant at the 95% confidence level. The Bland–Altman analysis demonstrated negligible systematic deviation between the two methods.

## 1. Introduction

Evaluation of digestive function in ruminants is essential for understanding nutrient utilization, feed efficiency, and overall animal productivity. Direct measurement of feed intake, particularly under grazing conditions, is challenging, while total fecal collection is labor-intensive and often impractical. Consequently, indirect approaches based on internal and external markers are widely applied to estimate fecal output, feed intake, and apparent dry matter digestibility [1]. External markers are typically added to the diet, while internal markers consist of indigestible components naturally present in the feed [2,3,4]. External markers include inert compounds such as chromic oxide (Cr_2_O_3_), titanium dioxide, n-alkanes, and lignin and lignin nanoparticles [4]. The marker should be quantitatively recovered in feces, not absorbed or retained in the gastrointestinal tract, and remain unaffected by digestive processes. Additionally, it should not interfere with the microbial population, should be non-toxic and cost-effective, readily measurable by analytical methods, and present only in small amounts in the original diet [2,4].

Chromic oxide has been widely used as an external marker in digestibility studies for several decades [4,5,6]. The compound is characterized by high chemical stability, resistance to enzymatic degradation, and nearly complete recovery in feces. The trivalent chromium present in its structure is poorly absorbed across cellular membranes and therefore exhibits limited bioavailability in the digestive tract. In contrast to Cr(VI), which is recognized as a strong oxidizing agent with toxic and carcinogenic effects under certain exposure conditions, there is no evidence that Cr(III) exhibits genotoxic or carcinogenic effects in vivo [7]. When administered at typical experimental doses, Cr_2_O_3_ does not adversely affect feed intake, physiological parameters, or animal welfare [8,9].

Accurate determination of chromium in feed and fecal samples is therefore essential in digestibility studies that use Cr_2_O_3_ as an inert marker. The analytical procedure typically involves mineralization of the organic matrix followed by spectrometric quantification of chromium in the resulting solution. However, reliable chromium determination in such matrices remains challenging because the sample digestion step is critical for achieving complete recovery. Chromic oxide is a highly stable and refractory compound with a compact corundum-type crystal structure characterized by strong Cr–O lattice bonds, which makes its quantitative dissolution difficult. Furthermore, Cr(III) is a kinetically inert d^3^ ion with slow ligand- and water-exchange rates. Consequently, the dissolution of Cr_2_O_3_ proceeds gradually from the crystal surface rather than uniformly throughout the lattice. Even under favorable conditions, this process remains kinetically slow. It may hinder complete chromium recovery from complex matrices such as fecal samples, which contain substantial amounts of organic and mineral components.

Various digestion procedures have been proposed for preparing feed and fecal samples. Most wet digestion methods rely on concentrated mineral acids, such as HNO_3_, HClO_4_, H_2_SO_4_, H_3_PO_4_, and HF, used in different combinations. In some protocols, additional reagents are introduced to facilitate oxidation or improve chromium recovery, including CaCl_2_ (as a releasing agent), Na_2_MoO_4_ as a catalyst, MnSO_4_, KBrO_3_, and H_2_O_2_ [10,11,12,13,14,15]. Dissolution of Cr_2_O_3_ can be achieved either by prolonged heating at temperatures around 200 °C or by applying optimized microwave-assisted digestion procedures. These treatments aim to convert chromium into a soluble form, either as Cr^3+^ or as hexavalent chromium in chromate or dichromate species, thereby enabling spectrometric determination.

When complete oxidation is achieved, the formed chromate or dichromate can be determined directly by colorimetry or after forming a colored complex with 1,5-diphenylcarbazide in an acidic medium [1,3,12,13].

With the development of analytical instrumentation, methods for the total determination of chromium in solution using flame atomic absorption spectrometry (FAAS) and ICP-OES have been widely implemented, providing better analytical reliability and improved accuracy [3,6,10,11,14,15]. Overall, the most critical step in these procedures remains sample dissolution, which is often labor-intensive, time-consuming, and requires large volumes of concentrated acids, increasing the risk of contamination and analyte loss. As a result, there is a growing interest in analytical strategies that simplify sample preparation while preserving adequate sensitivity and reliability.

Direct and slurry analyses of solid samples have proven to be valuable alternatives to methods based on wet digestion. Several authors have proposed direct analytical approaches for chromium determination, including near-infrared spectroscopy (NIRS) and portable X-ray fluorescence (p-XRF) methods [15,16]. These techniques aim to simplify sample preparation; however, when applied to complex biological matrices such as feed and fecal samples containing refractory Cr_2_O_3_, they exhibit some limitations. In such cases, sufficient analytical sensitivity and accuracy are not always achieved, as these methods may be affected by pronounced matrix effects and limited quantitative reliability.

Among these approaches, TXRF analysis [17] offers several advantages. The use of total reflection geometry significantly reduces matrix effects, while quantification based on a single internal standard improves analytical reliability compared to conventional XRF techniques [16,18]. TXRF has been widely applied for Cr determination in biological samples, as reviewed by several authors [17,19,20]. In addition, TXRF enables the direct analysis of finely dispersed suspensions, bypassing the requirement for complete dissolution of Cr_2_O_3_ and thereby minimizing extensive sample preparation. As an exemplary “green” analytical method, TXRF requires only milligram-level sample amounts and minimal sample pretreatment for suspension-based analysis. Furthermore, the measurements are rapid, automated, and characterized by low energy consumption, requiring no cooling water or carrier gases.

In this context, the aim of the present study was to develop and evaluate a simple and sensitive TXRF-based method for the determination of chromium in feed and fecal samples using suspension preparation. Due to the lack of suitable certified reference materials (CRMs), method verification was performed by comparison with an independent ICP-OES procedure following acid digestion of the samples. Very good agreement between the results obtained by both methods was demonstrated.

## 2. Results and Discussion

### 2.1. Optimization of TXRF Analytical Procedure for Chromium Determination in Feed and Fecal Samples

#### 2.1.1. Suspension Optimization and Internal Standardization

Key parameters for suspension preparation and measurement conditions were systematically evaluated and optimized to ensure accurate and reproducible TXRF results [21]. The proportions between ash mass, suspension volume, and internal standard concentration were adjusted to ensure stable measurements and reliable quantification.

Approximately 10 mg of ash was selected as a representative sample mass, allowing accurate weighing and good suspension homogeneity. Initial experiments using a suspension volume of 1 mL resulted in detector dead time values exceeding 30%, due to the high matrix load and increased absorption effects. Therefore, the samples were diluted to a final volume of 10 mL (1:10 dilution). Under these conditions, the chromium concentration in the suspensions ranged between approximately 14 and 25 mg L^−1^, and the detector dead time decreased to acceptable values (<10%), enabling high-quality spectral acquisition.

The preparation procedure, including ash grinding, vortex mixing, and ultrasonication followed by immediate aliquot withdrawal, was essential for obtaining homogeneous suspensions and reproducible analytical results. Particle aggregation strongly affects the distribution of solids within the thin residue layer formed after the deposited microdroplet dries on the sample carrier, potentially leading to variability in the measured elemental composition [21,22]. The particle size distribution of the ash suspensions was investigated to evaluate the potential influence of particle size and aggregation on the representativeness of the aliquots used for TXRF analysis (see Figure S1, Supplementary Material). Laser diffraction measurements revealed a bimodal particle size distribution, consisting of a fine fraction in the submicron range (~0.05–0.3 μm) and a dominant coarse fraction with a maximum in the range of approximately 15–25 μm. The substantial difference between the surface mean diameter (D_32_ ≈ 0.8 μm) and the volume mean diameter (D_43_ ≈ 22–23 μm) indicates a highly polydisperse system. This suggests that the coarse particles most likely correspond to aggregates formed from finer ash particles. To assess the efficiency of homogenization and the repeatability of both measurement and suspension preparation, two types of experiments were conducted: five parallel suspensions were prepared from a single sample, and three independent carriers were prepared from each suspension. The results showed RSD values between 2% and 4% for the independent carriers, confirming the repeatability of the deposition process, and RSD values between 4% and 7% for the parallel samples, indicating the repeatability of the overall procedure and the homogeneity of the suspensions. The good agreement between TXRF and ICP-OES results further demonstrates that the applied homogenization procedure provided adequate suspension stability, reliable aliquot sampling, and good overall method reproducibility (see Section 2.3).

Gallium was selected as the internal standard due to its high fluorescence yield and absence in the original samples, thereby preventing spectral interference. Its concentration was set at 10 mg L^−1^, resulting in a reference signal intensity comparable to that expected for chromium concentrations in the suspensions [21]. In addition, the large energy separation between the Cr Kα line (5.41 keV) and the Ga Kα line (9.25 keV) ensures reliable peak identification and prevents spectral overlap.

#### 2.1.2. Measurement Conditions and Carrier Selection

A deposition volume of 5 µL was selected to ensure that the dried residue remained within the effective excitation area of the carrier (~10 mm^2^) [23]. This volume provided adequate signal-to-noise ratios while maintaining stable measurement conditions. An exposure time of 200 s was found sufficient for quantification, as longer acquisition times (up to 1000 s) did not result in statistically significant improvements in the analytical results.

The influence of the carrier material was also investigated. For this purpose, suspensions of two feed samples and two randomly selected fecal samples were deposited onto acrylic and quartz carriers, and five parallel suspensions from each matrix were prepared and analyzed under identical experimental conditions. The differences in the determined chromium concentrations were negligible, and the deviations remained within the experimental uncertainty (see Section 2.3). Acrylic carriers were therefore selected for subsequent experiments due to their lower cost and the elimination of the rigorous acid-cleaning procedures required for quartz carriers.

#### 2.1.3. Spectral Characteristics and Repeatability

**Figure 1:**
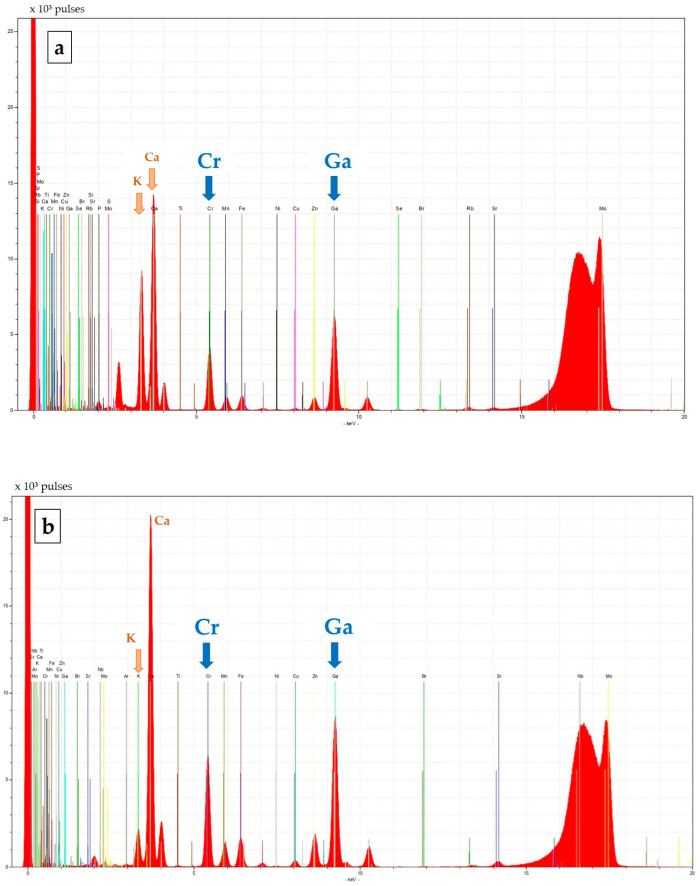
Representative TXRF spectra of (**a**) feed and (**b**) fecal suspension samples. The characteristic Kα peaks of Cr (5.41 keV) and Ga (9.25 keV, internal standard) are clearly resolved. Other detected elements include K and Ca as major components, as well as Fe, Mn, Zn, and Cu, along with trace elements originating from the sample matrix.

The recorded spectra exhibited well-resolved and distinct peaks with no significant interferences in the energy regions of interest (see Figure 1). The characteristic Kα lines of chromium and the internal standard gallium were clearly identifiable in spectra obtained from both feed and fecal samples. The absence of overlapping peaks in the vicinity of the Cr Kα line further confirms the selectivity of the TXRF determination in these matrices.

In addition to chromium, several matrix elements were detected, including K and Ca as major components, as well as Fe, Mn, Zn, and Cu. Differences in matrix composition were evident from the relative peak intensities, with a more pronounced K signal in the feed sample and a higher Ca signal in the fecal sample, reflecting expected variations between the two matrices. These observations confirm the suitability of TXRF for multi-elemental characterization while maintaining accurate chromium determination.

The precision of the method was evaluated by analyzing aliquots from suspensions prepared from three parallel samples, each deposited on a separate carrier. The resulting relative standard deviations (RSD) of 3–7% indicate good repeatability of the proposed TXRF method. Considering the heterogeneous nature of the ash suspensions and the micro-droplet deposition procedure, these values demonstrate satisfactory homogeneity and reproducible sample preparation. The addition of 0.01% Triton X-100 as a surfactant did not significantly influence the layer uniformity or the measured chromium concentrations, indicating that mechanical homogenization alone was sufficient.

**Figure 2:**
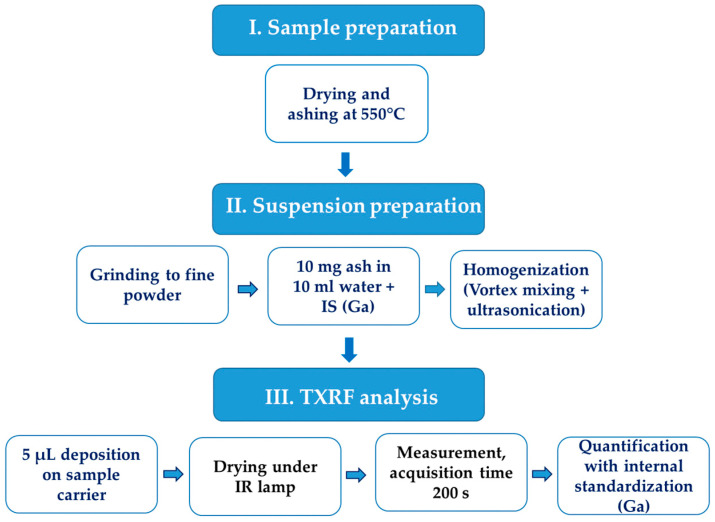
Schematic representation of the suspension-based TXRF procedure for chromium determination in feed and fecal samples.

A schematic representation of the developed suspension-based TXRF procedure is presented in Figure 2 to facilitate understanding of the analytical workflow.

### 2.2. Development of a Procedure for Total Cr Determination in Feed and Fecal Samples by ICP-OES

To verify the accuracy of the proposed TXRF method, the total chromium content in the investigated samples was also determined by ICP-OES after acid digestion.

Based on data from the literature and the requirements of ICP-OES analysis, a digestion mixture consisting of HNO_3_ acid and HClO_4_ acid was evaluated. Acids frequently reported in the literature, such as H_3_PO_4_ and H_2_SO_4_, were not investigated because they are generally unsuitable for ICP-OES measurements due to the formation of P–O and S–O species, which may interfere with plasma stability and spectral measurements. The use of catalysts was also avoided.

The sample mass used for analysis was evaluated in the range of 0.3–0.5 g. Experimental results indicated that the lower mass of 0.3 g provided more reliable digestion and improved dissolution of both the sample matrix and Cr_2_O_3_.

Since certified reference materials for fecal samples containing Cr_2_O_3_ are not available, the added–found approach was applied to evaluate the accuracy of the developed method. Model samples were prepared to assess the completeness of Cr_2_O_3_ dissolution. For this purpose, 0.005 g of solid Cr_2_O_3_ (0.0034 g as Cr) was analyzed directly, and control feed and fecal samples (0.3 g each) spiked with 0.005 g of solid Cr_2_O_3_ were also analyzed.

Two digestion procedures were evaluated: open-vessel digestion on a sand bath (see Section 3.4) and microwave-assisted digestion in closed vessels with a digestion mixture of 10 mL HNO_3+_ and 2 mL HClO_4_. The microwave digestion program was optimized in three steps: 130 °C for 10 min, 150 °C for 10 min, and 220 °C for 15 min. After cooling, the digested solutions were quantitatively transferred to 100 mL volumetric flasks and diluted to volume with deionized water.

Chromium was determined by ICP-OES using optimized instrumental parameters. Iron (Fe) and phosphorus (P), which are present in relatively high concentrations in the samples, have complex emission spectra with numerous lines in the UV region. Therefore, evaluation of potential spectral interferences was necessary, and several chromium emission lines were experimentally tested to select the most suitable line for Cr determination.

The spectral line at 267.716 nm is characterized by good sensitivity and performs well even in samples with high matrix loads. This line is commonly referenced in standardized methods for Cr determination because it is typically free from spectral interferences. The spectral line at 205.552 nm also exhibits high sensitivity. However, below 210 nm, the background continuum from Fe and P can be elevated, requiring careful baseline evaluation and background correction. Although less intense, the line at 357.869 nm is generally free from spectral interferences and is often used when UV lines are affected or might be affected by matrix interferences.

All three spectral lines were tested for Cr determination in solutions obtained after digestion of pure Cr_2_O_3_ and in representative control feed and fecal samples spiked with the same amount of Cr_2_O_3_. Results showed that both lines at 267.716 nm and 357.869 nm might be used for interference-free Cr determination. Results from recovery studies presented in Table 1 were obtained by ICP-OES measurements at 267.716 nm.

Evidently, open-vessel digestion yields recoveries of 95–102% for pure Cr_2_O_3_ and for feed and fecal samples. In contrast, microwave (MW) digestion is not efficient in this case, although it might normally be expected to work. Most likely, the high oxidative potential of hot HClO_4_ is the key factor enabling complete dissolution of Cr_2_O_3_. In microwave digestion procedures, HClO_4_ is typically used in a diluted mixture with HNO_3_, which likely explains the failure to dissolve Cr_2_O_3_ under those conditions.

The calibration approach was selected based on a comparison of the slopes of calibration curves obtained for feed/fecal samples and for standard solutions prepared in 0.1 M HNO_3_. The calculated slopes were consistent within the limits of random error, indicating that external calibration can be used for Cr quantification.

The analytical procedure developed for total Cr determination in feed and fecal samples using ICP-OES is based on the defined, optimized conditions and is described in Section 3.4. Analytical figures of merit were determined after analysis of five parallel samples of feed and fecal samples according to the developed analytical procedure (Section 3.4). The relative standard deviation of Cr results ranged from 4% to 7% for both feed and fecal samples. The limit of detection and limit of quantification, calculated based on the 3σ and 10σ criteria, are 5 µg g^−1^ and 8 µg g^−1^, respectively, for total Cr. These are recalculated as 16 µg g^−1^ and 24 µg g^−1^ for Cr_2_O_3_, which fulfill the requirements for digestibility studies. Although CRM is not available for full validation of this procedure, it is believed that complete dissolution of Cr_2_O_3_, as confirmed by the added/found method and reliable ICP-OES measurements, ensured accurate results.

### 2.3. Method Verification

The most reliable approach for validating the TXRF analytical method is to analyze an appropriate certified reference material (CRM). However, in the present study, a suitable CRM for the investigated matrix was not available. Therefore, the accuracy of the developed TXRF method was confirmed through comparison with an independent analytical technique.

**Figure 3:**
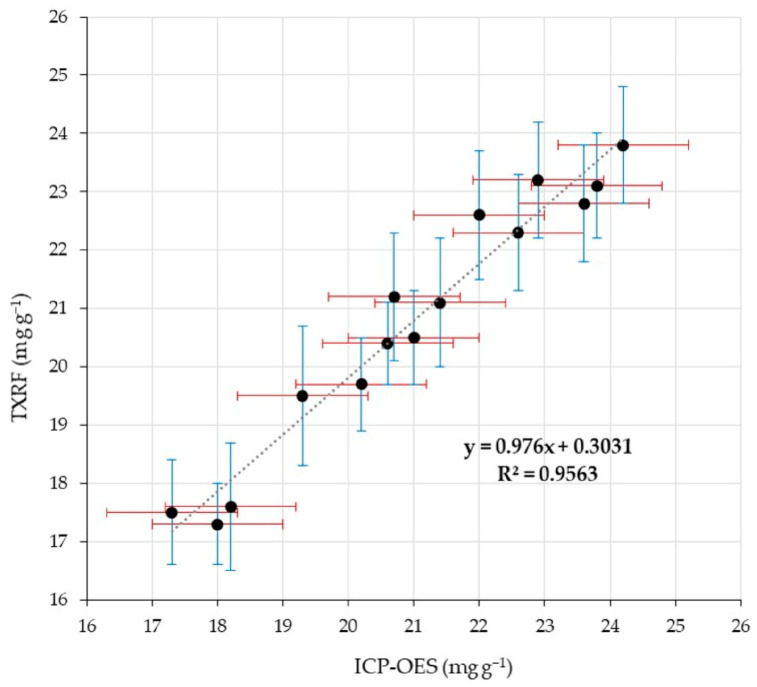
Comparison of chromium concentrations determined by TXRF and ICP-OES in fecal samples (n = 15). Error bars represent the standard deviation of replicate measurements. The dashed line indicates the linear regression between both methods.

For this purpose, the chromium concentrations determined by the developed TXRF method in two replicate feed samples and in a set of fecal samples were compared with results obtained by an independently developed ICP-OES method following sample acid digestion. The ICP-OES method developed in the frame of this study is considered accurate and suitable as a reference method, provided that complete Cr_2_O_3_ dissolution is confirmed and reliable Cr measurement is achieved. Comparative results are presented in Table 2 and graphically illustrated in Figure 3. Notably, the standard deviations of the results obtained by both analytical methods are very similar. The similarity in measurement variability strengthens the reliability of the statistical comparison and supports the validity of conclusions regarding the performance of the developed TXRF method.

As an initial step in the comparison, a scatter plot was used to visualize the relationship between paired measurements obtained by the two techniques across the entire concentration range. Each data point in the scatter plot represents the mean value of three parallel measurements for the respective sample (Figure 2). As shown, the slope of the regression line is 0.9767, indicating good agreement between the TXRF and ICP-OES concentrations. Furthermore, the calculated correlation coefficient (R^2^ = 0.9559) demonstrates a strong linear relationship between the two sets of measurements, suggesting that both analytical methods produce consistent results over the studied concentration range.

To further evaluate potential systematic differences, a paired t-test was applied to the dataset. The agreement between the two datasets was evaluated using a paired *t*-test. The calculated *t* value (−0.197) is much lower than the critical *t* value (2.024) at the 95% confidence level, and the corresponding two-tailed *p*-value (0.845) is significantly higher than 0.05 (see Table S1, Supplementary Material). This indicates that there is no statistically significant difference between the two sets of results. This statistical outcome confirms that the developed TXRF method yields results comparable to those obtained with the reference ICP-OES method.

In addition to regression analysis and hypothesis testing, method agreement was further assessed by calculating the relative bias (%) and recovery (%) for each sample (Table 2). The obtained values indicate that the TXRF results are in good agreement with those obtained by ICP-OES, with no pronounced systematic deviation observed.

**Figure 4:**
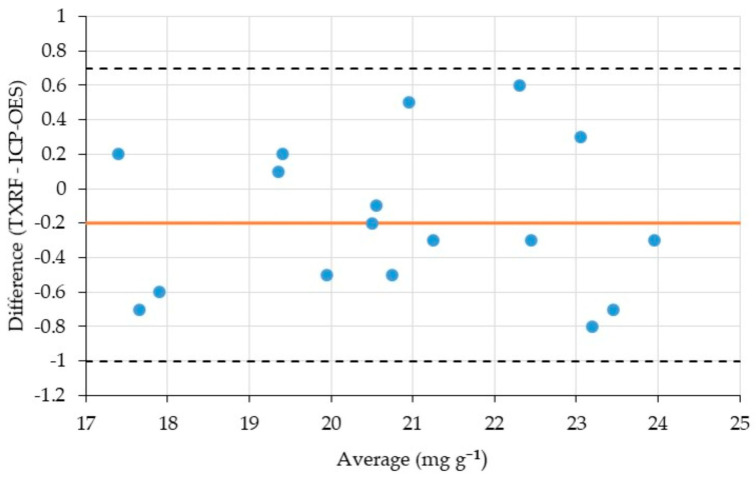
Bland–Altman plot showing the agreement between TXRF and ICP-OES for Cr determination in fecal samples. The central line represents the mean bias, and the dashed lines correspond to the limits of agreement (±1.96 SD).

A more detailed evaluation of method agreement was performed using the Bland–Altman approach, based on the differences between paired fecal sample measurements (Figure 4). The mean bias (−0.2 mg g^−1^) indicates negligible systematic deviation between TXRF and ICP-OES. Meanwhile, the observed standard deviation (0.4 mg g^−1^) is consistent with typical analytical variability for suspension-based and digestion-based measurements. The limits of agreement (−1.0 to +0.7 mg g^−1^) fall within a practically acceptable range, confirming that the TXRF method yields results comparable to those of the reference ICP-OES procedure for the analyzed samples.

It should be noted that the TXRF analysis of two replicate feed samples resulted in chromium recoveries of 97–98% relative to the expected chromium content based on the addition of 0.18% Cr_2_O_3_ to the feed. This agreement between expected and experimentally determined values provides additional support for the accuracy of the developed TXRF procedure.

Overall, the combined evaluation using regression analysis, paired t-tests, bias estimation, and limits of agreement demonstrates that the developed TXRF method is capable of producing reliable and accurate results for the determination of chromium in fecal and feed samples. The obtained Cr concentrations in fecal samples (17–25 mg g^−1^ ash, corresponding to 25–36.5 mg g^−1^ as Cr_2_O_3_) and in feed samples (~14–15 mg g^−1^ ash) are consistent with values typically reported in the literature for ashed biological matrices. These concentration levels are representative of digestibility studies conducted under steady-state conditions, such as during a 6-day feeding period, when the external marker has reached a stable excretion rate. This indicates that the proposed method operates within a concentration range relevant for routine applications in animal nutrition studies.

Furthermore, unlike ICP-OES, which requires complete acid digestion of samples, TXRF allows direct analysis of finely dispersed suspensions, simplifying sample preparation. This reduces reagent consumption, minimizes potential contamination, and shortens the overall analysis time.

## 3. Materials and Methods

### 3.1. Materials, Reagents, and Instruments

All reagents were of analytical-reagent grade, and all aqueous solutions were prepared using high-purity water (Millipore Corp., Milford, MA, USA). A certified chromium stock standard solution (TraceCERT^®^, 1 g L^−1^ in HNO_3_, Merck KGaA, Darmstadt, Germany) was used to prepare working calibration solutions at concentrations of 0.5–100 mg L^−1^ in 0.1 M HNO_3_.

A gallium stock solution (1000 mg L^−1^, Merck, Darmstadt, Germany) was used as an internal standard for TXRF measurements.

Drying of samples was performed in a ventilated laboratory oven, while ash preparation was conducted in a muffle furnace (Linn High Therm, Hirschbach, Germany). Suspensions were homogenized using a Vortex mixer (PV-1, Grant Instruments, Royston, UK) and an ultrasonic bath (Elmasonic P70H, Elma Schmidbauer GmbH, Singen, Germany). Mass measurements were performed using an analytical balance (Kern, Frankfurt, Germany).

TXRF measurements were carried out using an S2 PICOFOX 400 spectrometer (Bruker Nano GmbH, Berlin, Germany) equipped with a Mo anode X-ray tube and a multilayer monochromator, providing monochromatic radiation at 17.5 keV. Fluorescence signals were detected using a silicon drift detector (SDD, active area 30 mm^2^, energy resolution < 149 eV). Samples were deposited onto fused-silica or acrylic carriers (30 mm in diameter, 3 mm thick). Spectral data were evaluated using SPECTRA 7 software.

Chromium concentrations were determined using a Prodigy 7 ICP-OES instrument (Teledyne Leeman Labs, Hudson, NH, USA). The measurements were performed at 267.716 nm, the wavelength of the most sensitive chromium emission line, free of spectral interferences. Plasma emission was observed in radial view. Quantification was carried out using external calibration with aqueous standard solutions.

### 3.2. Feeding Experiments and Sample Preparation

The feeding experiment was conducted with early-weaned Lacone lambs during February and March 2025 on a private livestock farm in Popovitsa, Plovdiv region, Bulgaria. To assess compound feed intake, chromic oxide was included in the diet as an external marker at 0.18% of feed dry matter according to standard procedures used in ruminant nutrition studies. The compound was thoroughly blended with the vitamin–mineral premix, sodium chloride, and flavoring agents using a micro-ingredient dosing system. The resulting homogeneous premix was subsequently introduced into a 1-ton capacity mixer, where all concentrate components were mixed for 20 min.

Fecal samples were collected individually from each lamb on the sixth day of the experimental period using collection diapers applied for 24 h. The samples were dried in a ventilated oven at 65 °C for 24 h, ground in a laboratory mill equipped with a 1 mm sieve. Portions of approximately 3 g of as-prepared fecal samples and 3 g of dried feed samples were gradually ashed in a muffle furnace using a stepwise temperature program up to 550 °C for 8 h until white or light gray ash was obtained. After cooling in a desiccator, the ash was weighed and stored for subsequent analysis.

### 3.3. Suspension Preparation and TXRF Measurements

Prior to TXRF analysis, the loose ash was manually ground in an agate mortar for 15 min to obtain a fine, homogeneous powder.

Approximately 10 mg of the powdered ash was accurately weighed (±0.0001 g) into a 15 mL polyethylene tube. A 100 µL aliquot of Ga stock solution (1000 mg L^−1^) was added as an internal standard, and the volume was adjusted to 10 mL with ultrapure water, resulting in a final Ga concentration of 10 mg L^−1^.

The suspensions were homogenized by vortex mixing for 30 s, followed by ultrasonication in an ultrasonic bath for 15 min to disrupt possible micro-agglomerates. Immediately after a final 5-s vortexing step, a 5 µL aliquot of the suspension was pipetted onto an acrylic sample carrier. The deposited samples were dried under an infrared lamp prior to measurement. TXRF spectra were recorded using an S2 PICOFOX 400 spectrometer with an acquisition time of 200 s. Three independent replicates were prepared from each suspension to ensure analytical reproducibility.

Chromium quantification was carried out by internal standardization using Ga (10 mg L^−1^). Detector gain calibration was performed using the As Kα line (10.53 keV). The analyte concentration and the limit of detection (LOD) were automatically calculated from the net peak intensities according to the following expressions:

(1) $$C_{i}\;=\;\frac{C_{IS}\times N_{i}\times S_{IS}}{N_{IS}\times S_{i}}$$

(2) $${LOD}_{i}=\frac{3\times C_{i}\times \surd N_{BG}}{N_{i}}$$

where I denotes the analyte element (Cr); $N_{i}$ and *N_IS_* are the net peak intensities of Cr and Ga, respectively; *S_I_* and *S_IS_* are their relative sensitivities; *C_IS_* is the concentration of the internal standard; and *N_BG_* represents the background count rate.

### 3.4. Sample Preparation and ICP-OES Measurements

Approximately 0.3 g of the sample was accurately weighed using an analytical balance and transferred to a 50 mL glass beaker. Accordingly, 10 mL of concentrated HNO_3_ was added, and the mixture was allowed to stand for approximately 8 h at room temperature. The beaker was then placed on a sand bath at approximately 120 °C, covered with a watch glass, and the sample was digested for about 4 h. After digestion, the watch glass was removed, and the solution was evaporated to near dryness. After cooling, 2 mL of concentrated HClO_4_ (caution: HClO_4_ forms explosive mixtures with organic matter; it should be used after preliminary oxidation of organic matter with HNO3) was added, and the mixture was heated to approximately 180 °C until near dryness was again achieved. The residue was allowed to cool, after which approximately 10 mL of 1 M HNO_3_ was added. The resulting suspension was quantitatively transferred to a 100 mL volumetric flask and diluted to volume with distilled water. After approximately 3 h, the suspension settled at the bottom of the flask, producing a clear supernatant suitable for ICP-OES analysis. Filtration of the sample was not required.

Three parallel preparations were carried out for each sample. Chromium quantification was performed using the external calibration method, with a calibration curve constructed from standard solutions prepared from a certified chromium stock solution. The calibration range was selected according to the expected chromium concentration in the digested samples and typically covered 0.05–100 mg L^−1^.

## 4. Conclusions

The results demonstrate that TXRF represents a reliable and efficient technique for the determination of chromium in lamb’s feed and fecal samples when Cr_2_O_3_ is used as an indigestible marker. Comparative results for the proposed approach and published methods are presented in Table S2 of the Supplementary Material. The optimized suspension-based TXRF method provides accurate and reproducible results while offering several practical advantages, including minimal sample preparation without complete digestion, straightforward quantification using a single internal standard, low reagent consumption, and rapid analysis of small sample quantities. The obtained reproducibility and limits of quantification satisfy the analytical requirements of digestibility studies. Owing to its simplicity, robustness, and high sample throughput, the proposed method represents a practical and effective alternative to conventional techniques for routine monitoring of chromium markers in animal nutrition and digestibility research.

## Figures and Tables

**Table 1:** Recovery studies (mean ± SD), three parallel determinations.

Sample	Parameter	Digestion	
		Sand Bath	MW Digestion
Cr_2_O_3_	Added, mg	5.0	5.0
	Found, mg	4.9 ± 0.2	0.9 ± 0.2
	Recovery (%)	98 ± 4	18 ± 5
Feed spiked with Cr_2_O_3_	Determined	<LOQ	<LOQ
	Added, mg	5.1	5.1
	Found, mg	5.2 ± 0.3	2.1 ± 0.2
	Recovery, %	102 ± 5	42 ± 4
Feces spiked with Cr_2_O_3_	Determined	<LOQ	<LOQ
	Added, mg	4.9	4.9
	Found, mg	4.8 ± 0.1	2.8 ± 0.2
	Recovery, %	98 ± 2	57 ± 4

**Table 2:** Comparison of Cr concentrations determined by TXRF and ICP-OES in feed (F) and fecal (S) ash samples. Results are presented as mean ± SD (three parallel samples analyzed by ICP-OES and three parallel suspensions prepared for TXRF). Relative bias (%) and recovery (%) were calculated to evaluate systematic differences and agreement between the two methods.

Sample	TXRF (mg g^−1^)	ICP-OES (mg g^−1^)	Bias (%)	Recovery (%)
F1	14.4 ± 0.9	14.7 ± 0.9	−2.0	98.4
F1 (quartz)	14.5 ± 1.0	14.7 ± 0.9	−1.4	98.6
F2	14.5 ± 0.9	14.7 ± 0.8	−1.4	98.6
F2 (quartz)	14.5 ± 0.8	14.7 ± 0.8	−1.4	98.6
S1	20.4 ± 0.7	20.6 ± 0.9	−1.9	98.1
S1 (quartz)	20.5 ± 0.8	20.6 ± 0.9	−1.4	98.6
S2	19.5 ± 1.2	19.3 ± 1.1	1.0	101.0
S2 (quartz)	19.4 ± 0.9	19.3 ± 0.7	0.5	100.5
S3	21.2 ± 1.1	20.7 ± 1.2	2.4	102.4
S4	19.7 ± 0.8	20.2 ± 1.2	2.5	97.5
S5	22.6 ± 1.1	22.0 ± 1.0	2.7	102.7
S6	22.3 ± 1.0	22.6 ± 1.2	−1.3	98.7
S7	20.5 ± 0.8	21.0 ± 0.8	−2.4	97.6
S8	17.3 ± 0.7	18.0 ± 0.8	−3.9	96.1
S9	22.8 ± 1.0	23.6 ± 1.1	−3.4	96.6
S10	21.1 ± 1.1	21.4 ± 1.2	−1.4	98.6
S11	17.6 ± 1.1	18.2 ± 0.9	−3.3	96.7
S12	17.5 ± 0.9	17.3 ± 0.7	1.2	101.2
S13	23.8 ± 1.0	24.1 ± 1.2	−1.7	98.3
S14	23.2 ± 1.0	22.9 ± 1.1	1.3	101.3
S15	23.1 ± 0.9	23.8 ± 1.0	−2.9	97.1

Samples marked as “quartz” were measured using quartz sample carriers, while the remaining samples were analyzed on acrylic carriers. The comparison between quartz and acrylic carries was based on five parallel samples. The limits of detection (LODs) were automatically calculated for each TXRF spectrum, with mean values ranging from 1 to 4 µg g^−1^ ash.

## Data Availability

The original contributions presented in this study are included in the article. Further inquiries can be directed to the corresponding author.

## Supplementary Materials

The following supporting information can be downloaded at https://www.mdpi.com/article/10.3390/molecules31091522/s1. Figure S1: Particle size distribution; Table S1: Results of the paired t-test for comparison of TXRF and ICP-OES results; Table S2: Comparative overview of analytical methods used for Cr_2_O_3_ determination in feed and fecal samples. References [10,11,12,13,14,15,16,24,25,26,27] are cited in the supplementary materials.
