# Supplementary material for: Development and Verification of a Suspension-Based TXRF Method for Chromium Determination in Feed and Fecal Samples Containing Chromic Oxide as an External Digestibility Marker
Source: Molecules. 2026 May 3;31(9):1522. doi: 10.3390/molecules31091522 (PMC13165053; doi:10.3390/molecules31091522)
Supplement: Supplementary file 1 [file molecules-31-01522-s001.zip › molecules-4234648-supplementary.pdf]

Supplementary materials

Development and verification of a suspension-based TXRF method for chromium determination in feed and fecal samples containing chromic oxide as an external digestibility marker

Christina Tzvetkova, Lidia Ivanova, Albena Detcheva, Antonina Kovacheva, Miroslav Simeonov and Irina Karadjova*

Corresponding author email: imkaradjova@svr.igic.bas.bg

Figures

Figure S1, Section 2.1.1

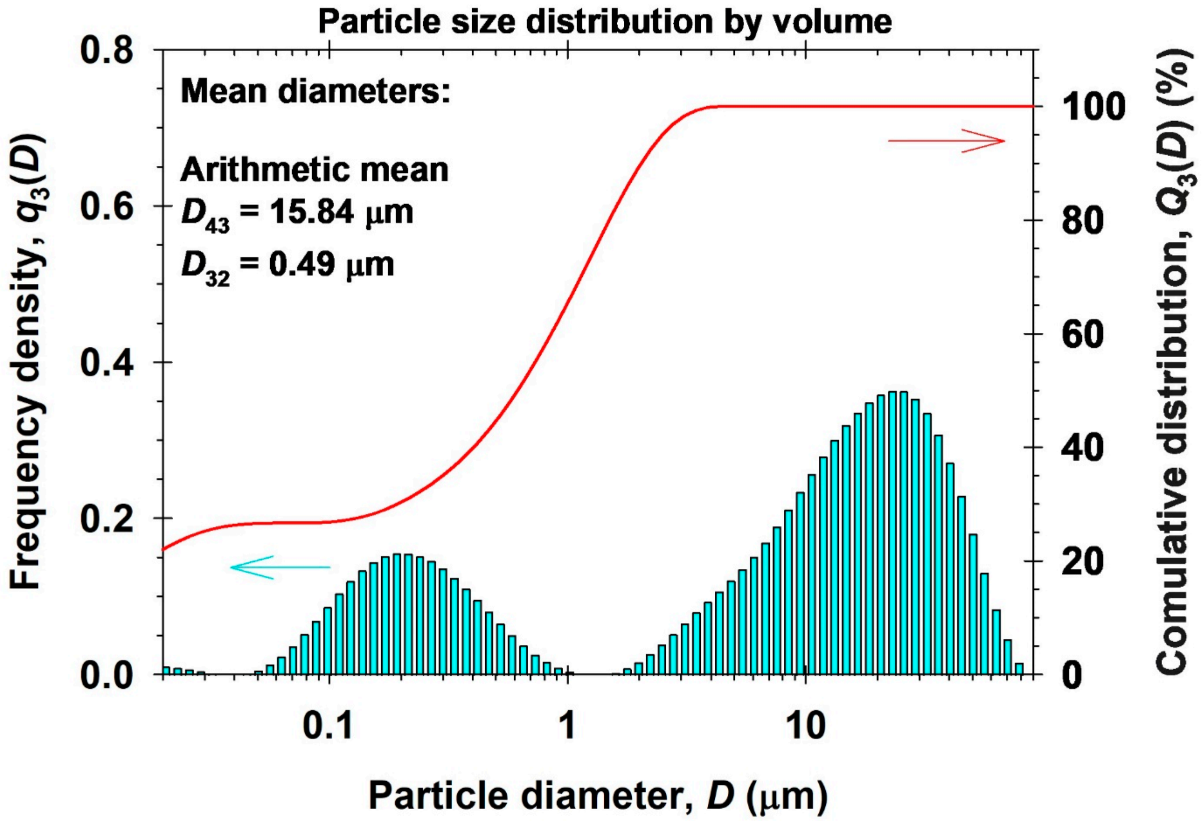

Figure S1. Particle size distribution

Laser Particle Sizer ANALYSETTE 22 NanoTec (FRITSCH GmbH)

Theory: Mie Liquid Refractive Index (water at 20 ^{0}C) = 1.3328

Solid Refractive Index = 1.5 or 1.45

Tables

Table S1, section 2.3

Table 1S Results of the paired *t*-test for comparison of TXRF and ICP-OES results.

Parameter	Results	
Mean	19,8	19,985
Variance	8,812632	8,808711
Observations	20	20
Pooled Variance	8,810671	
Hypothesized Mean Difference	0	
df	38	
t Stat	-0,19709	
P(T<=t) one-tail	0,422403	
t Critical one-tail	1,685954	
P(T<=t) two-tail	0,844807	
t Critical two-tail	2,024394	

Table S2, section 4

Table 2S Comparative overview of analytical methods used for Cr_2O_3 determination in feed and fecal samples

Method	Sample preparation	LOD*	Advantages	Limitations	Ref.
FAAS	Drying, ashing, acid digestion	76 µg L^{-1} [5]	Robust, widely used	Time-consuming, high acid consumption	[1-6]
UV–Vis spectrophotometry	Drying, ashing, acid digestion, oxidation	0.98 µg g^{-1} [7]	Simple, low cost	Matrix effects, limited accuracy	[1, 3, 7, 8]
ICP-OES	Digestion (microwave/ open vessel)	0.6-23 µg g^{-1} [9, 10]<br>5 µg g^{-1} (this study)	High sensitivity, multi-element capability	Complex preparation, hazardous reagents	[9, 10]<br>this study
GFAAS (slurry)	Fine grinding, suspension preparation	0.8 µg L^{-1}	Very high sensitivity, no digestion	Sensitive to preparation conditions	[5]
p-XRF / NIRS	Minimal preparation	not reported	Rapid,<br>non-destructive	Poor quantitative accuracy, strong matrix effects	[10, 11]
**TXRF (this study)**	Suspension of ash,<br>no digestion	1-4 µg g^{-1}	Minimal preparation, no reagent consumption, reduced matrix effects	Requires fine grinding and efficient homogenization	this study

*The reported LOD values are taken from the respective literature sources and are provided for general comparison only. Direct comparison between methods should be made with caution, as these values depend strongly on sample preparation procedures, matrix composition, analyte form, and instrumental conditions. In addition, differences in units and data treatment may further limit comparability. Furthermore, LOD values are not consistently reported in the literature for all methods.

References

1. Souza, N.K.P.; Detmann, E.; Pina, D.S.; Valadares Filho, S.C.; Sampaio, C.B.; Queiroz, A.C.; Veloso, C.M. Evaluation of chromium concentration in cattle feces using different acid digestion and spectrophotometric quantification techniques. *Arq. Bras. Med. Vet. Zootec.* **2013**, *65*, 1472–1482.
2. Rocha, G.C.; Palma, M.N.N.; Detmann, E.; Valadares Filho, S.C. Evaluation of acid digestion techniques to estimate chro-mium contents in cattle feces. *Pesqui. Agropecu. Bras.* **2015**, *50*, 92–95.
3. Guzman-Cedillo, A.E.; Corona, L.; Castrejon-Pineda, F.; Rosiles-Martínez, R.; Gonzalez-Ronquillo, M. Evaluation of chro-mium oxide and titanium dioxide as inert markers for calculating apparent digestibility in sheep. *J. Appl. Anim. Res.* **2017**, *45*, 275–279.
4. de Melo, L.P.; Rennó, L.N.; Detmann, E.; Paulino, M.F.; da Silva Júnior, R.G.; Ortega, R.M.; Moreno, D.S. Effect of supple-mentation plans and frequency on performance and metabolic responses of grazing pregnant beef heifers. *Vet. Sci.* **2024**, *11*, 506.
5. Silva, F.A.; Padilha, C.C.F.; Pezzato, L.E.; Barros, M.M.; Padilha, P.M. Determination of chromium by GFAAS in slurries of fish feces to estimate the apparent digestibility of nutrients in feed used in pisciculture. *Talanta* **2006**, *69*, 1025–1030.
6. Yiakoulaki, M.D.; Papadoyannis, I.N.; Nastis, A.S. Determination of marker chromic oxide in faeces of grazing goats on Mediterranean shrublands by AAS. *Anim. Feed Sci. Technol.* **1997**, *67*, 163–168.
7. Ospina, A.T.; Zapata, J.A.; Gil Gonzalez, J.H.; Giraldo, L.; Valencia, D.M. Colorimetric method as alternative to chromium (III) quantification in cattle feces. *Acta Sci. Anim. Sci.* **2020**, *42*, e48096.
8. Mioto da Costa, M.C.; Ítavo, L.C.V.; Ferreira Ítavo, C.C.B.; Dias, A.M.; Petit, H.V.; Reis, F.A.; Gomes, R.C.; Leal, E.S.; Niwa, M.V.G.; de Moraes, G.J. Evaluation of internal and external markers to estimate faecal output and feed intake in sheep fed fresh forage. *Anim. Prod. Sci.* **2018**, *59*, 741–748.
9. Levine, K.E.; Stout, M.D.; Ross, G.T.; Essader, A.S.; Perlmutter, J.M.; Grohse, P.M.; Fernando, R.A.; Lang, M.; Collins, B.J. Validation of a method for the determination of total chromium in rat feces by inductively coupled plasma optical emis-sion spectrometry. *Anal. Lett.* **2009**, *42*, 2729–2746.
10. Sierra-Alarcón, A.M.; Parra-Forero, D.M.; Calvo-Salamanca, A.M.; Durán-Cruz, E.N.; Gualdrón-Duarte, L.B.; Ariza-Nieto, C.; Ortiz-Cuadros, R.; Mayorga-Mogollón, O.L. Rapid determination of indigestible NDF and the markers chromium oxide and titanium dioxide of supplement, forages and faecal samples by ICP-OES and near-infrared reflectance spectroscopy. *Discov. Animals* **2025**, *2*, 95.
11. Barnett, M.C.; Forster, N.A.; Ray, G.A.; Li, L.; Guppy, C.N.; Hegarty, R.S. Using portable X-ray fluorescence (pXRF) to determine fecal concentrations of non-absorbable digesta kinetic and digestibility markers in sheep and cattle. Anim. Feed Sci. Technol. 2016, 212, 35-41
